# 24-Epibrassinolide Ameliorates Endogenous Hormone Levels to Enhance Low-Temperature Stress Tolerance in Cucumber Seedlings

**DOI:** 10.3390/ijms19092497

**Published:** 2018-08-24

**Authors:** Ali Anwar, Longqiang Bai, Li Miao, Yumei Liu, Shuzhen Li, Xianchang Yu, Yansu Li

**Affiliations:** 1 The Institute of Vegetables and Flowers, Chinese Academy of Agricultural Sciences, Beijing 100081, China; dr.ali_ivf@yahoo.com (A.A.); bailongqiang01@163.com (L.B.); happymml@163.com (L.M.); sd.liuyumei@163.com (Y.L.); limengfbj@126.com (S.L.); 2 College of Agricultural and Biological Engineering, Heze University, Heze 274015, China

**Keywords:** EBR, chlorophyll, antioxidant, hormones, cucumber, low-temperature stress

## Abstract

Phytohormone biosynthesis and accumulation are essential for plant growth and development and stress responses. Here, we investigated the effects of 24-epibrassinolide (EBR) on physiological and biochemical mechanisms in cucumber leaves under low-temperature stress. The cucumber seedlings were exposed to treatments as follows: NT (normal temperature, 26 °C/18 °C day/night), and three low-temperature (12 °C/8 °C day/night) treatments: CK (low-temperature stress); EBR (low-temperature and 0.1 μM EBR); and BZR (low-temperature and 4 μM BZR, a specific EBR biosynthesis inhibitor). The results indicated that low-temperature stress proportionately decreased cucumber seedling growth and the strong seedling index, chlorophyll (Chl) content, photosynthetic capacity, and antioxidant enzyme activities, while increasing reactive oxygen species (ROS) and malondialdehyde (MDA) contents, hormone levels, and EBR biosynthesis gene expression level. However, EBR treatments significantly enhanced cucumber seedling growth and the strong seedling index, chlorophyll content, photosynthetic capacity, activities of antioxidant enzymes, the cell membrane stability, and endogenous hormones, and upregulated EBR biosynthesis gene expression level, while decreasing ROS and the MDA content. Based on these results, it can be concluded that exogenous EBR regulates endogenous hormones by activating at the transcript level EBR biosynthetic genes, which increases antioxidant enzyme capacity levels and reduces the overproduction of ROS and MDA, protecting chlorophyll and photosynthetic machinery, thus improving cucumber seedling growth.

## 1. Introduction

During their life cycles, plants are challenged by many kinds of biotic and abiotic stresses, which influence their growth and productivity. To survive environmental stresses, plants have evolved physiological and biochemical resistance mechanisms [1]. Low-temperature stress hampers plant growth and development, resulting in yield reductions, especially in vegetable crops, such as cucumber, tomato, and pepper [2,3,4]. Greenhouse vegetable production is very common in the world during winter cultivation, though frequent sudden temperature drops occur, which can lead to serious plant damage. Low temperatures lead to reduced plant growth, flower drop, reduced yield, and economic losses in vegetable crops. Previous studies reported that chilling stress hampers plant growth and development by decreasing enzymatic activities, increasing the accumulation of reactive oxygen species (ROS), damaging membrane stability, reducing chlorophyll (Chl) biosynthesis, and impairing the photosynthetic machinery [5,6].

Brassinosteroids are a group of phytohormones that regulate many biological and developmental processes in plants, from germination to fruit development [7,8]. Thousands of genes regulated by 24-epibrassinolide (EBR) are involved in plant growth and development [4]. Exogenous applications of EBR can affect a variety of physiological processes and increase plant tolerance to different kinds of stresses [2,3], such as low temperatures, heavy metals, and drought stress [9,10,11]. 24-epibrassinolide is the most active synthetic analog of the EBR family, and it improves low-temperature stress tolerance in tomato, pepper, cucumber, and eggplant [3,4,9,12]. The functional mechanisms of EBR in plant-stress responses have been reported in various studies [13,14]. It promotes tolerance to stresses, including heat, cold, drought, and salinity, as indicated by observed correlation with expression of stress marker genes, including heat-shock protein and cold-responsive genes [15,16,17]. Under stress conditions, EBR increases biosynthesis of Chl and the photosynthetic machinery, as well as activity of stress-tolerance enzymes [2,18,19]. 

The interactions among, and regulation of, plant hormones are important in acclimation to low-temperature stress [20,21]. These phytohormones regulate various developmental process under stress conditions [8,16]. Exogenous EBR regulated endogenous abscisic acid is an important plant hormone, and its accumulation is a key factor in controlling downstream responses that are essential for plants to adapt different kinds of stresses [16,21,22]. Increases in cytokinin levels enhance many physiological process, including cell division, chloroplast development, seed germination, leaf senescence, nutrient mobilization, gene expression regulation, and ethylene biosynthesis under stress conditions [23,24,25]. Auxin is produced in young leaves and is involved in many growth and developmental processes, including cell division, phototropism, gravitropism, and phytohormone regulation to increase stress tolerance [26]. Gibberellins (GAs) are produced in roots and play important roles in growth, promoting cell division, increasing nitrogen metabolism, and promoting stem and root elongation, as well as reducing stress effects [27]. Regulation by phytohormones is very important for plant growth, and each hormone plays a dynamic role in the induction of stress tolerance [8]. EBR enhances the plant antioxidant defense system and increases plant tolerance, but interactions of EBR with endogenous phytohormones, including GA, indole-3-propionic acid (iPA), ascorbic acid (ABA), jasmonic acid (JA), indole acetic acid (IAA), salicylic acid (SA), and zeatin riboside (ZR), need to be further explored. The correlations of EBR with plant growth and the defense system, including activity of superoxide dismutase (SOD), peroxide (POD), catalase (CAT), glutathione reductase (GR), and ascorbate peroxide (APX), also requires additional study.

Cucumber (*Cucumis sativus*) is an economically important crop worldwide. It is a summer crop with an optimum growth temperature of 26 °C/18 °C day/night [27]. Cucumber can grow during the winter in solar greenhouses, where exposure to low temperatures is the main cause of reduced growth and yield [10]. The goals of this study were to investigate the physiological mechanisms behind cucumber seedling responses to exogenous EBR applications under low-temperature stress and to explore the role of EBR in the regulation of endogenous phytohormones and its correlation with cucumber growth under low-temperature stress.

## 2. Results

### 2.1. Effects of EBR Treatments on Cucumber Seedling Growth and the Strong Seedling Index under Low-Temperature Stress Conditions

**Figure 1:**
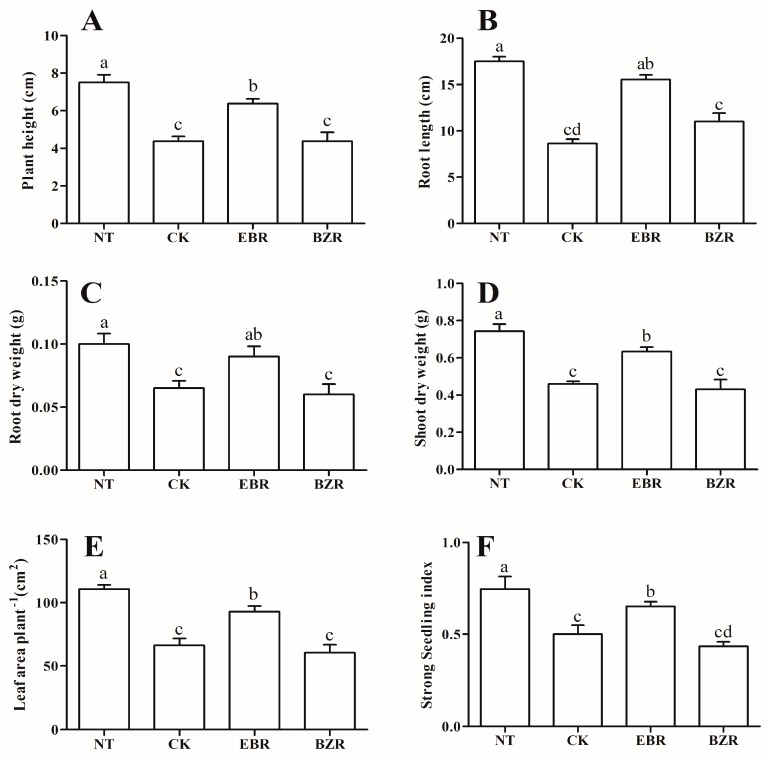
Effect of EBR application and low-temperature stress on cucumber seedling growth ((**A**) plant height, (**B**) root length, (**C**) root dry weight, (**D**) shoot dry weight, (**E**) leaf area) and (**F**) the strong seedling index. Different letters indicate significant differences at *p* < 0.05.

Abiotic stress negatively affected plant growth, leading to reduced yield and yield components [23]. Plant height, root length, root and shoot dry weight, and leaf area of cucumber seedlings when exposed to low-temperature stress experienced reductions of 41.66%, 50.71%, 37.09%, 36.55% and 36.75%, respectively, with reductions of 15.55%, 11.23%, 12.90%, 10.21% and 11.30% for EBR treated seedlings, both as compared to NT (Normal Temperature; 26 °C/18 °C, day/night) (Figure 1). The root dry weight increased by 38.46% after EBR application and decreased by 7.69% after BZR (Brassinazole, a specific EBR biosynthesis inhibitor) application, compared with the CK treatment (Figure 1C). Similarly, shoot dry weight and leaf area per plant also increased by 41.52% and 40.22%, respectively, after EBR application and decreased after BZR application, compared with the CK treatment (Figure 1D,E). The strong seedling index increased by 33.2% in the EBR treatment and decreased by 13.42% after the BZR treatment, compared with the CK treatment (Figure 1F). The strong seeding index was significantly higher in NT, and decreased by 27.91%, 4.16% and 37.54% in CK, EBR, and BZR respectively. Exogenous EBR application reduced the harmful effects of low-temperature stress and increased the health of cucumber seedlings. 

### 2.2. Effects of EBR Treatments on Chlorophyll Content under Low-Temperature Stress Conditions

Low-temperature stress induced a significant decrease in the Chl a, Chl b, Chl a+b, and carotenoid contents, but increased the Chl a/b ratio as compared with the EBR treatment (Table 1). However, the Chl a, Chl b, Chl a+b, and carotenoid contents were increased by exogenous EBR application in cucumber leaves under low-temperature stress conditions, resulting in increases of 23.08%, 42.85%, 19.51% and 26.08%, respectively. Compared with NT, Chl a, Chl b, Chl a+b, and carotenoid contents decreased 8.86%, 42.30%, 7.55% and 17.92% in EBR treated seedlings, but 25.94%, 59.62%, 22.64% and 34.91%, in the CK treatment, indicating that EBR reduced the harmful effects of low-temperature stress and enhanced chlorophyll accumulation (Table 1). There were no significant differences in the Chl contents between the CK and BZR treatments. 

### 2.3. Effects of EBR Treatments on Photosynthesis under Low-temperature Stress Conditions

Cucumber seedlings treated with EBR exhibited significantly increased photosynthetic capacity, including elevated values of net photosynthesis (Pn), stomatal conductance (Gs), intercellular CO_2_ concentration (Ci), and transpiration rate (Tr) when compared with the Ck and BZR treatments (Table 2). Under low-temperature stress, the EBR treatment increased Pn, Gs, Ci, and Tr by 20.46%, 23.07%, 36.61% and 65.23%, as compared with the CK treatment. Compared with NT, Pn, Gs, Ci and Tr decreased in the EBR treatment by 16.07%, 11.11%, 2.7% and 11.93% and by 30.33%, 27.77%, 28.78% and 46.70% in the CK treatment, respectively (Table 2). The BZR treatment also negatively affected photosynthetic capacity under low-temperature stress when compared with the NT treatment. The compression of the photosynthetic parameters under EBR and CK showed that EBR reduces the effect of low temperature stress. Exogenous EBR application enhanced the photosynthetic capacity under low-temperature stress. 

### 2.4. Effects of EBR Treatments on Antioxidant Enzyme Activity Levels

**Figure 2:**
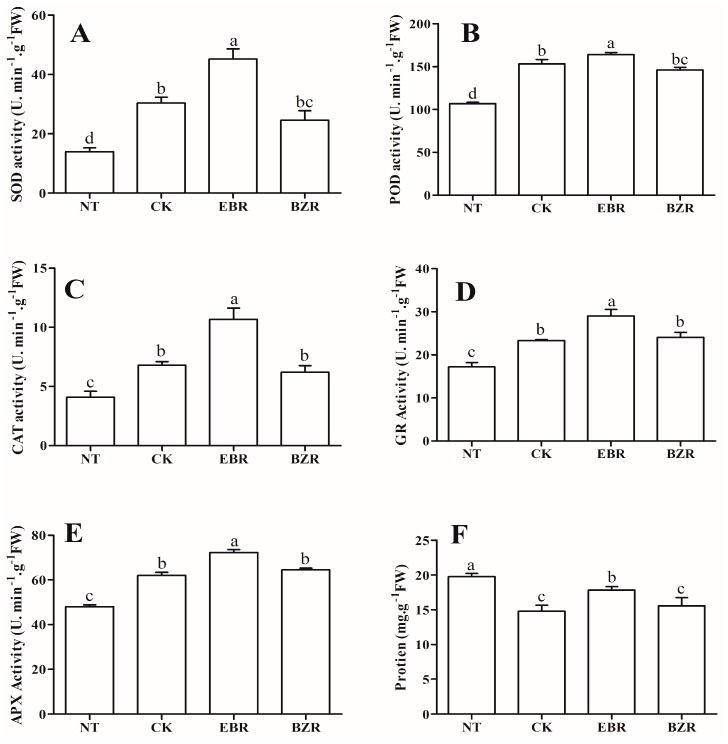
Effect of EBR application and low-temperature stress on antioxidant enzyme activities (**A**) superoxide dismutase (SOD), (**B**) peroxide (POD), (**C**) catalase (CAT), (**D**) glutathione reductase (GR), (**E**) ascorbate peroxide (APX), (**F**) protein) in cucumber seedlings. Different letters indicate significant differences at *p* < 0.05.

The antioxidant enzyme activity (SOD, POD, GR, CAT and APX) levels were significantly increased in EBR, as compared to the NT, CK and BZR treatments, as measured 7 days after exposure to low-temperature stress, as presented in Figure 2. The antioxidant enzymes activity levels were the same in BZR and CK, but significantly higher than NT. Exogenous EBR increased SOD, POD, GR, CAT, and APX activity by 116.18%, 38.68%, 62.23%, 148.61% and 48.40%, and the CK treatment enhanced their activity by 45.20%, 29.46%, 30.55%, 58.43% and 27.29%, respectively, all as compared to NT. The BZR and CK treatments did not differ detectably from each other, but in both, activity was enhanced significantly as compared with NT. The antioxidant enzyme activity was significantly lower in NT, but this treatment enhanced protein content over that of the low-temperature stress treatments (CK, EBR, and BZR). EBR may regulate the plant defense system to reduce the adverse effect of low-temperature stress.

### 2.5. Effects of EBR Treatments on ROS, MDA, and CMS

**Figure 3:**
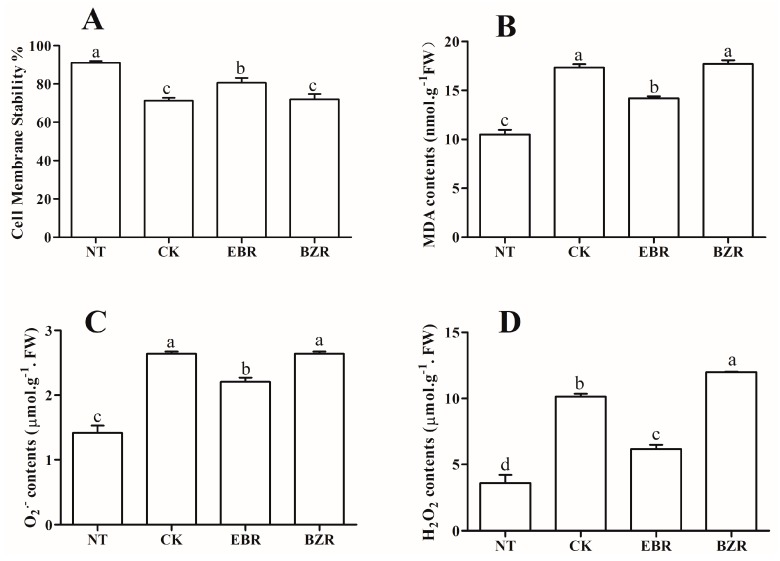
Effects of EBR on (**A**) Cell membrane stability %, (**B**) MDA, (**C**) O_2_^·−^, and (**D**) H_2_O_2_ in cucumber seedlings under low temperature stress. Different letters indicate significant differences at *p* < 0.05.

ROS (H_2_O_2_, O_2_^·−^) acts as a secondary messenger during low-temperature stress and causes oxidative stress when overproduced. H_2_O_2_, O_2_^·−^, and MDA contents were significantly enhanced under low-temperature stress by 181.56%, 86.57% and 64.97%, respectively, as compared to NT, and reduced by exogenous EBR application by 71.30%, 5.87% and 35.10%, as compared to CK (Figure 3). The lowest H_2_O_2_, O_2_^·−^, and MDA contents were found in NT, followed by EBR, while the maximum was reported in BZR followed by CK, although there was no significant difference between BZR and CK. These findings showed that EBR stabilized ROS and MDA production under low-temperature stress. CMS was measured in cucumber seedlings exposed to low-temperature stress for 7 days (Figure 3A), and the maximum CMS value occurred in NT followed by the EBR treatment, significantly higher than in the BZR and CK treatments. The EBR treatment increased the CMS by 14.02% relative to the CK treatment, but was statistically similar to the CK and BZR treatments. Protein contents were significantly higher in NT followed significantly by EBR, but the minimum occurred in the CK and BZR treatments (Figure 2F). These findings indicate that cucumber seedlings are more stable under low-temperature stress when treated with EBR when compared with the CK treatment. 

### 2.6. Effects of EBR Treatments on Hormone Contents under Low-Temperature Stress Conditions

**Figure 4:**
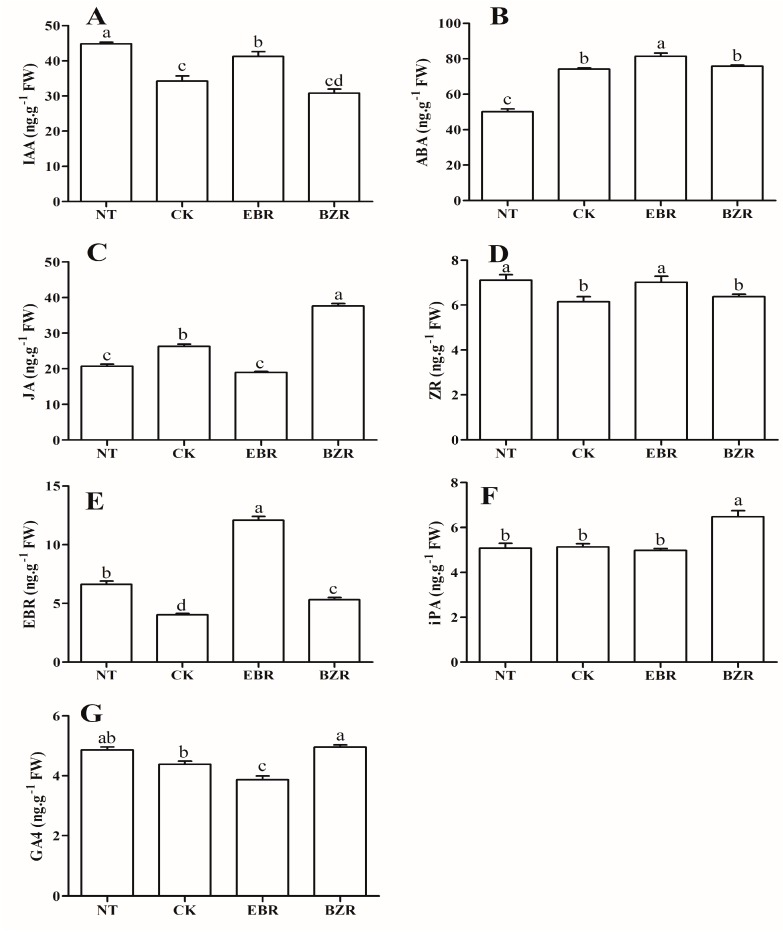
Effect of EBR on endogenous hormone regulation under low-temperature stress in cucumber seedlings. (**A**) Indol-3-acetic acid (IAA); (**B**) Ascorbic acid (ABA); (**C**) Jasmonic acid (JA); (**D**) Zeatin riboside (ZR); (**E**) Brassinosteroid (EBR); (**F**) Isopentenyl adenosine (iPA); (**G**) Gibberellin A4 (GA4). Different letters indicate significant differences at *p* < 0.05.

Exogenous EBR applications significantly increased the plant hormone content in leaves of cucumber seedlings exposed to low-temperature stress (Figure 4). Low-temperature stress reduced the leaf IAA content, as compared to the NT and EBR treatments (Figure 4A). Additionally, the IAA content increased by 20.49% in the EBR treatment but decreased by 10.01% in the BZR treatment, and by 30.92% in NT treatment, when compared with the CK treatment. Low temperature significantly reduced the ABA content, but it was enhanced by exogenous EBR application. The ABA contents were increased by 9.72% and 7.33% in the EBR treatment, as compared to CK and BZR treatments, respectively, whereas the NT treatment resulted in the lowest ABA contents (Figure 4B). The EBR content increased in the EBR treatment by 200% and 126.91% compared with the CK and BZR treatments, respectively. Endogenous EBR contents increased under NT at 64.64% and 24.55%, as compared to CK and BZR, respectively, but decreased by 82.21% as compared to EBR (Figure 4E). The ZR content also significantly increased in the EBR and NT treatments, but decreased with the CK and BZR treatments (Figure 4D). Low-temperature stress (CK) increased the JA (27.91%) content in leaves of cucumber seedlings, BZR treatment increased the JA content (49.58%), and NT also increased JA (9.33%), compared with the EBR treatment (Figure 4C). EBR significantly negatively affected GA4 accumulations, which increased by 11.77% in the CK treatment, with the BZR treatment increased the GA4 content 21.91% beyond that of the CK treatment, while NT and BZR did not differ detectably (Figure 4G). In addition, the iPA content was statistically equivalent after the EBR, CK, and NT treatments as well, but it increased 23.07% after the BZR treatment (Figure 4F).

### 2.7. EBR Biosynthesis Gene Expression Levels under Low-Temperature Stress after EBR Application

**Figure 5:**
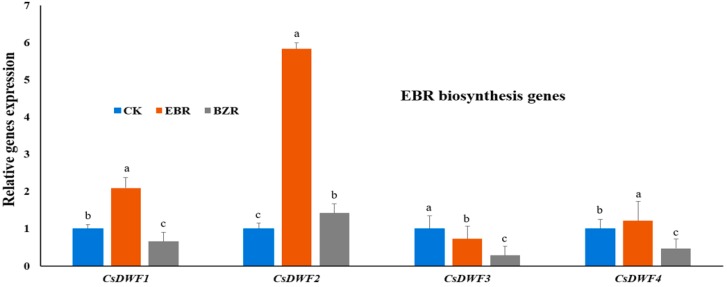
EBR regulates transcript levels of EBR biosynthesis genes under low temperature stress in cucumber seedlings. Different letters indicate significant differences at *p* < 0.05.

*DWF*s are key EBR biosynthesis genes in plants [9,10,13]. Expression levels were investigated in cucumber seedlings exposed to low-temperature stress (Figure 5). Exogenous EBR significantly increased the expression levels of EBR biosynthesis-related genes *CsDWF1*, *CsDWF2*, and *CsDWF4*, but *CsDWF3* was downregulated. Low-temperature stress substantially reduced the expression levels of *CsDWF1*, *CsDWF2*, and *CsDWF4*, but up-regulated *CsDWF3* gene expression level, as compared to EBR. Additionally, BZR-treated seedlings down-regulated *CsDWF1*, *CsDWF3*, *CsDWF4* genes expression levels, but up-regulate *CsDWF2*, as compared to CK. The expression level of *CsDWF3* gene was down-regulated by exogenous EBR and BZR application, but significantly up-regulated in CK. The down-regulated *CsDWF* genes allowed for significant incremental increases in defense-related enzyme activities, which resulted in increased plant growth (both fresh and dry weight) under low-temperature stress, suggesting that EBR induced low-temperature stress tolerance.

## 3. Discussion

EBR is a member of a class of steroidal plant growth regulators that can increase stress tolerance and promote growth and yield [20]. In the present study, exogenous EBR significantly enhanced cucumber seedling growth under low-temperature stress conditions (Figure 1). EBR can reduce the harmful effects of low temperature. The results corroborate those of earlier studies, which reported that exogenous EBR increases plant tolerance to chilling and salt stress [12,28].

Chlorophyll (Chl) is commonly used as an indicator of chloroplast development and photosynthetic proficiency [1,3]. Chl degradation is accelerated by various abiotic stresses and ultimately reduces the photosynthetic capacity, as observed in many plant species [29,30,31]. Additionally, Chl degradation also depends upon the duration and intensity of the stress [29,31]. Here, low-temperature stress reduced the Chl contents, but exogenous EBR application significantly increased the total Chl content (Chl a, Chl b, and carotenoid), but reduced the Chl a/b ratio under low-temperature stress conditions (Table 1) [3,32,33]. Chl a contents decreased by 25.95% in the CK treatment and by 20.89% in BZR-treated seedlings, while Chl b decreased by 59.62% and 57.69% in the CK and BZR treatments, respectively. These findings suggest that Chl b is very sensitive to stress and easily degraded, and subsequently, the Chl a/b ratio increased after the CK and BZR treatments (Table 1). Furthermore, the degradation of Chl a in EBR (8.86%) was significantly lower than in CK (25.95%) and BZR (20.89%), compared to NT, and the same trends were found for Chl b and carotenoids. These findings help to elucidate the dynamic role of EBR in response to stress and to explain why exogenous EBR enhanced seedling growth (Figure 1) [3]. The increase in Chl content may result from direct or indirect involvement of EBR in stimulating Chl biosynthetic enzyme activity levels or in protecting them from stress [10], as well as from stimulating Chl biosynthesis-related gene expression levels in plants under stress conditions [4,19,31,33].

Photosynthesis is directly related to plant growth and yield [5,33]. As presented in Table 2, the EBR treatment increased the photosynthetic capacity under low-temperature stress conditions. The photosynthetic capacity in EBR treated seedlings was significantly higher, as compared to the CK and BZR treatments, but decreased when compared with NT. These findings are supported by previous studies, which reported that EBR can increase photosynthetic capacity under abiotic stress [1,2,3,4,5]. EBR promotes photosynthesis in cucumber by positively regulating the synthesis and activation of different photosynthesis-related enzymes, including Rubisco, and increasing expression of genes (*rca*, *rbcS* and *rbcl*) related to photosynthesis [3,30]. A transcriptome analysis revealed that EBR upregulates thousands of genes, including 29 genes that are related to photosynthesis in pepper plants under chilling stress [4]. Thus, EBR appears to increase photosynthesis under low-temperature stress conditions in cucumber seedlings to promote growth.

Exposure to environmental stress leads to the overproduction of ROS in plants, causing damage to proteins, lipids, carbohydrates, and DNA, which ultimately results in oxidative stress, reduced cell membrane stability (CMS), and ultimately to cell death [34]. Plants possess antioxidant defense systems protective against oxidative damage during abiotic stress (salt, chilling, high temperature, and drought stresses) [34,35]. Exogenous EBR application increased antioxidant enzyme activities (SOD, POD, CAT, GR, and APX) as well as protein content under low-temperature stress conditions (Figure 2), and reduced MDA and ROS (H_2_O_2_ and O_2_^·−^) contents (Figure 3). Increased MDA and ROS production damages metabolism through oxidative injury to plant cells, chloroplasts, mitochondria, chlorophyll, and the photosynthetic apparatus, and decreases antioxidant enzyme activities [34,36]. Our results reveal lower MDA and ROS contents and higher activity levels of antioxidant enzymes (Figure 2) following exogenous EBR application. This implies that EBR can alleviate membrane damage when cucumber seedlings are exposed to low-temperature stress. The results corroborate those of previous studies, in which EBR-treated plants showed more tolerance than did untreated plants to low-temperature and weak-light stress [3], to salt stress in *Perennial ryegrass* [37], and to salt, polyethylene glycol, and low-temperature stresses in *Solanum melongena* [2]. These studies suggested that increasing antioxidant enzyme activities and the transcript levels of defense-related genes by exogenous EBR applications occurred as a result of increased hormone levels [14,19]. Thus, the EBR-related increase in low-temperature stress tolerance may be closely related to its activation of the cucumber antioxidant defense system, stabilizing the overproduction of ROS and MDA and resulting in an increased membrane stability index [31,35,38]. Cucumber seedlings treated with EBR showed increased growth compared with untreated plants (Figure 1), which supports a role for EBR in plant response to low-temperature stress.

Phytohormones are essential in plant growth and development, and for increasing stress tolerance [25,39,40]. Phytohormones, such as EBR, ABA, IAA, JA, SA, and GA, regulate defense systems and increase plant tolerance levels to different kinds of stresses [8,16,41,42]. Our results indicated that exogenous EBR application increased the accumulated levels of ABA, IAA, ZR, and EBR under low-temperature stress conditions in cucumber, while the levels of JA and GA4 decreased (Figure 4). The iPA level was the same after the CK and EBR treatments. The results corroborate those of earlier studies, in which exposure to EBR increased plant hormone biosynthesis under stress conditions [16]. EBR can induce the SA-perceptive pathway and ABA-dependent pathway in response to stress [4,15,16]. The auxin, JA, and SA signal transduction pathways appear to act synergistically with EBR to induce stress tolerance [21,26,42]. 

ABA, which is important for adaptation to various kinds of stress [15], increased after EBR treatments under low-temperature stress (Figure 4B). Interactions between ABA and EBR regulate the expression of genes involved in many biological processes, such as primary root and hypocotyl elongation, seed germination, stomatal closure, and responses to environmental stresses [23,40,43]. EBR-related mutants increase the sensitivity to the inhibitory effects of ABA during seed germination [25,39]. Additionally, an exogenous EBR application increases water-stress tolerance by increasing ABA biosynthesis [22]. A microarray analysis showed that ABA regulates a large number of EBR-responsive genes [25], indicating a strong correlation between EBR and ABA, which might result in EBR enhancing stress tolerance. Our results are supported by previous studies, which reported that exogenous EBR increases chilling tolerance by enhancing the ABA contents [14,28].

IAA plays a crucial role in the coordination of many growth and development processes throughout the life cycle of a plant [26]. Interactions between EBR and IAA regulate many aspects of plant growth and development [4,26,28]. Furthermore, auxin regulates EBR biosynthetic genes (such as *DWF4*, *CPD*, and *BIN2*) [21,44,45], whereas *BIN2* mediates auxin response factor 2 phosphorylation and leads to the up-regulation of EBR gene expression and the promotion of auxin response factor activities, which are responsible for IAA signaling and EBR–IAA synergistic interactions [24,46]. As presented in Figure 4A, EBR increased the IAA content under low-temperature stress conditions [28]. In *Arabidopsis*, auxin synergistically functions as a biosynthetic signal for EBR, and exogenous auxin applications significantly enhanced the expression of *DWF4* and endogenous EBR levels in *DWF4pro*: *GUS* plants, which supports our findings [44].

Zeatin riboside (ZR; a cytokinin) and EBR interactions are important for plant growth and development [24]. ZR stimulates EBR accumulation as indicated by the interaction between ZR and EBR in *Chlorella vulgaris*, in which ZR increased endogenous EBR content several-fold. ZR regulates EBR biosynthetic (*DWF4* and *DWF5*) and signaling (*BRI*, *BZR1*, and *BAK1*) genes [13,24]. Here, exposure to EBR increased the ZR content, suggesting that interaction between EBR and ZR contributes to increasing low-temperature tolerance (Figure 4D). The EBR content was several-fold higher after EBR treatments (Figure 4E) than after CK treatments, suggesting that exogenously applied EBR induced low-temperature stress tolerance by activating specific EBR signal-transduction pathways [45], while the iPA content remained equivalent to that of CK, when compared with the EBR treatment (Figure 4F). The JA and GA4 contents were increased by low-temperature stress but reduced by the application of exogenous EBR (Figure 4C,G). The same mechanism was also reported in earlier studies, in which *ABA5* and *RGA* targeted the SOMNUS (*SOM*) gene under heat-stress conditions to regulate germination, while SOM expression was regulated by ABA5 and deactivated by GA. Additionally, it was reported that SOM represses seed germination through the simultaneous promotion of ABA and inhibition of GA biosynthesis [20,22,23,25,30,39]. Thus, under stress conditions, ABA (Figure 4B) may affect GA biosynthesis (Figure 4G), resulting in decreases in the GA4 and JA contents, while the ABA content increases under low-temperature stress conditions.

Hormonal regulation and biosynthesis are important aspects of plant growth. In this study, we reported that EBR biosynthetic genes are upregulated by exogenous EBR applications. These results are corroborated by those of earlier studies, which revealed that EBR treatments increased the levels of *DWF1*, *DWF2*, and *DWF4* transcripts, which encode enzymes that synthesize bioactive EBR, while BRZ (specific EBR inhibiter) treatments and low-temperature stress decreased their levels (Figure 5) [36,47]. Our findings are also supported by earlier studies in which *AtDWF4* overexpression in *Arabidopsis* seedlings increased cold-stress tolerance and germination [16], and exogenous EBR induced the expression of genes involved in stress resistance (*SOD*, *POD*, heat-shock protein 70, and cold-responsive) in cucumber [36,48,49]. Similarly, in this study cucumber seedlings treated with EBR had enhanced levels of EBR biosynthetic gene transcripts under low-temperature conditions (Figure 5), which resulted a significant incremental increase in growth (Figure 1) [50,51]. 

Here, we presented physiological (cucumber seedling growth, root and shoot dry weights, leaf area, strong seedling index, Chl content, and photosynthetic parameters) and biochemical (antioxidant enzyme activities, MDA and O_2_^·−^ contents, CMS, hormonal regulation, and transcript levels of EBR biosynthetic genes) mechanisms of EBR under low-temperature stress conditions, which revealed a dynamic role for EBR in low-temperature stress tolerance (Figure 5). To explore the role of EBR, we also compared CK and EBR results with NT, which indicated that EBR enhances plant growth by activating the plant defense system and regulating hormone biosynthesis. 

## 4. Material and Methods

### 4.1. Plant Material and Growth Conditions

The experiment was conducted from March to November 2017 in controlled growth chambers at the Institute of Vegetables and Flowers, Chinese Academy of Agricultural Sciences, Beijing, China. Cucumber (*Cucumis sativus* L. Cv. Zhongnong 26) obtained from the Institute of Vegetables and Flowers, Chinese Academy of Agricultural Sciences, was selected as the experimental material. After germination on moist gauze in a Petri dish in the dark at 28 °C, the sprouting seeds were transplanted to a seedling tray (32-hole plate) filled with soil medium. They were placed at 28 °C day/18 °C night with 70–75% relative humidity, and 300–350 μmol·m^−2^·s^−1^ photosynthetically active radiation was provided for 14 h. When cotyledons were fully expanded, same size seedlings were transplanted to plastic containers (34 cm × 26 cm × 12 cm; six seedlings per container) filled with half strength Hoagland’s nutrient solution. They were allowed to acclimate for 10 days before being assigned to treatments.

### 4.2. Treatments and Sampling

At the fully-expanded 1st leaf stage, cucumber seedlings were divided into four groups: NT cucumber seedlings were exposed to normal temperature (26 °C/18 °C, day/night), while other three groups were exposed to low temperatures, and designated as Control (CK), EBR (treated with 0.1 μM EBR), and BZR (4 μM BZR; Brassinazole, a specific EBR biosynthesis inhibitor). Treatments were sprayed on cucumber seedling leaves until they were completely wet at 3-day intervals. The relatively moderate 0.1 μM concentration of EBR is very effective as previously reported [36] and was therefore used in this experiment. The CK treatments were treated with the same concentration of ethanol. The EBR stock solution was prepared by dissolving EBR in ethanol, while BZR was dissolved in Dimethyl Sulfoxide (DMSO), and solutions were stored at 4 °C. Tween-20, 0.02% *v*/*v* was used as the surfactant at application time. The experiment was repeated three times, with three pots (containers) per replicate for each treatment. The treated seedlings were exposed to a low-temperature regime of 12 °C day/8 °C night at a relative humidity of 70–75% and 300–350 μmol·m^−2^·s^−1^·photosynthetically active radiation, with a photoperiod of 14 h. The seedlings were exposed to low-temperature stress for 7 days. The fully-expanded 2nd and 3rd leaves were sampled, immediately flash-frozen in liquid nitrogen, and stored at −80 °C until required for various analyses. 

### 4.3. Measurement of Growth Parameters

The plant height and root length were determined using a ruler, while the hypocotyl diameter was determined using a digital Vernier caliper. To determine fresh weights, roots and shoots were separated and weighed, and the same plants were also used for leaf area determinations. The plants were placed in an oven at 105 °C for 30 min and then dried to a constant weight at 75 °C. These plants were weighed to record plant dry weights. The strong seedling index was determined as follows:

(1) $$\mathrm{Strong}\;\mathrm{Seedling}\;\mathrm{Index}\;=(\frac{\mathrm{Hypocotyl}\;\mathrm{Diameter}}{\mathrm{Plant}\;\mathrm{Height}\;}\;+\;\frac{\mathrm{Root}\;\mathrm{Dry}\;\mathrm{Weight}\;}{\mathrm{Shoot}\;\mathrm{Dry}\;\mathrm{Weight}})\times \;\mathrm{Total}\;\mathrm{Dry}\;\mathrm{Weight}$$

### 4.4. Measurement of Chlorophyll Content

The total chlorophyll (Chl) content was determined by extraction in 95% ethanol and was measured using a spectrophotometer; the absorbance levels were measured at 665, 649 and 470 nm, following established methods of Wu et al. [33].

### 4.5. Measurement of Gas-Exchange Parameters

The net photosynthesis (Pn), stomatal conductance (Gs), transpiration rate (Tr), and intercellular CO_2_ concentration (Ci) of the 2nd fully expanded leaves were measured using a portable photosynthesis system (LI-6400XT, Lincoln, NE, USA (www.licor.com)). Five plant of the same size leaves were selected form each treatments from the controlled growth chambers between 11 a.m. and 12 p.m. to ensure maximum photosynthesis.

### 4.6. Leaf Antioxidant Enzyme Activity Levels and Malondialdehyde (MDA) Contents

Fresh leaves (0.5 g) were ground using a pre-chilled pestle and mortar in 4 mL ice-cold 0.05 mol/L sodium phosphate buffer (pH 7.8). The homogenate was centrifuged at 10,500× *g* for 20 min at 4 °C. The supernatant was used to determine the antioxidant activity levels. SOD activity was determined by measuring its ability to inhibit the photochemical reduction of nitro blue tetrazolium; absorbance was read at 560 nm [52]. CAT activity was measured as the decline in the absorbance at 240 nm resulting from a decrease in the extinction of H_2_O_2_. POD activity was measured as the increase in absorbance at 470 nm. APX activity was measured by the increase in absorbance at 290 nm as ASA was oxidized. GR activity was measured using the rate of decrease in the absorbance of NADPH at 340 nm [44], while MDA and protein contents were measured by the TBA and Bradford methods [38].

### 4.7. Determination of the H_2_O_2_ and O_2_^·−^ Content

The H_2_O_2_ and O_2_^·−^ contents were determined using an assay kit (COMINBIO; http://www.cominbio.com) and using UV-1800 spectrophotometer (SHIMADZU; Gifu, Japan), following the manufacturer’s instructions [53]. The absorbance was recorded at 415 and 530 nm, respectively. 

### 4.8. Cell Membrane Stability (CMS)

The CMS of cucumber seedlings was estimated using the following formula

CMS (%) = [1 − (T1/T2)]/[1 − (C1/C2)] × 100(2)

where T indicates treatment, C indicates control, and 1 and 2 indicate the initial and final conductance measurements [35].

### 4.9. Leaf Hormone Extractions and Quantifications

Leaf hormone (ABA, IAA, GA4, JA, ZR, iPA and EBR) contents were determined using enzyme-linked immunosorbent assay technology at the College of Agronomy and Biotechnology, China Agriculture University, Beijing, China [54]. The fresh leaf samples (0.5 g) were homogenized in liquid nitrogen and extracted in ice-cold methanol (80%, *v*/*v*) with 1 mmol/L butylated hydroxytoluene and kept at 4 °C overnight. The samples were centrifuged for 20 min at 10,000× *g* at 4 °C. The extracts were filtered through a C_18_ Sep-Pak cartridge (Waters Corp., Milford, MA, USA) and dried with liquid nitrogen. The residues were dissolved in 0.01 mol/L PBS (Phosphate Buffered Saline) (pH 7.4) to determine the hormone levels. Microtitration plates (Nunc, Sigma, Santa Clara, CA, USA) were coated with synthetic ABA, IAA, GA4, JA, ZR, iPA, and EBR ovalbumin conjugates in 50 mmol/L NaHCO_3_ buffer (pH 9.6) and kept at 37 °C overnight. A 10 mg/mL ovalbumin solution was added to each well to block nonspecific binding. The samples were again incubated for 30 min at 37 °C, and then desired hormone standards and antibodies were added and samples were again incubated for 45 min at 37 °C. Antibodies against the hormones were obtained [47] and then horseradish peroxidase-labelled goat antirabbit immunoglobulin was added to each well and incubated for 1 h at 37 °C. The buffer enzyme substrate was added, and enzyme reactions were allowed to proceed in the dark at 37 °C for 15 min. They were stopped by the addition of 3 mol/L H_2_SO_4_, and the absorbance levels were recorded at 490 nm. The hormone contents were calculated by adding known amounts of standard hormones to the split extracts.

### 4.10. Quantitative Real-Time PCR (qRT-PCR)

The total RNA was extracted from cucumber leaves using the RNAprep Pure Kit (For Plants) (TIANGEN, Beijing, China), according to the instructions, and the first-strand cDNA was synthesized using a FastQuant RT kit (TIANGEN) according to the manufacturer’s instructions and used as the template in the amplification assay. qRT-PCR and melting curve analyses were performed following the instructions of the SuperReal pre Mix plus (SYBER Green) Kit (TIANGEN) on an Applied BioSystems 7500 Real Time PCR system (Applied BioSystems, Model No. 401511) with specific primers (Table 3). The final volume of the reaction was 20 μL, containing 10 μL 2× SYBER premix Ex Taq TMII, 0.4 μL 50× ROX Reference Dye II, 0.4 μL of each primer, and 8.2 μL of a 5× dilution of the cDNA template. The thermal cycling conditions were as follows: 95 °C for 15 min, 40 cycles of 95 °C for 10 s, 60 °C for 20 s, 72 °C for 32 s, and a final extension at 72 °C for 5 min. For each qPCR experiment, a no cDNA-template control was included to ensure that the reagents and RNA samples were free of genomic DNA contamination. The amplifications were performed in 96-well plates. For relative quantification, *Actin* was detected as an internal reference, and the 2^−∆∆*C*t^ method was used. The Primer Premier 5 software was used to design primers. To confirm the specificity of the amplification reactions, melting curve analyses were performed using the method recommended by the manufacturer of the Stratagene Mx3000p system to identify putative nonspecific PCR products.

### 4.11. Statistical Analyses

There were four independent biological replications for each treatment, and the whole experiment was repeated three times. The data were statistically analyzed using analysis of variance (ANOVA), and treatments were compared using the LSD test (*p* < 0.05) using Statistix 8.1 software (www.statistix.com).

## 5. Conclusions

**Figure 6:**
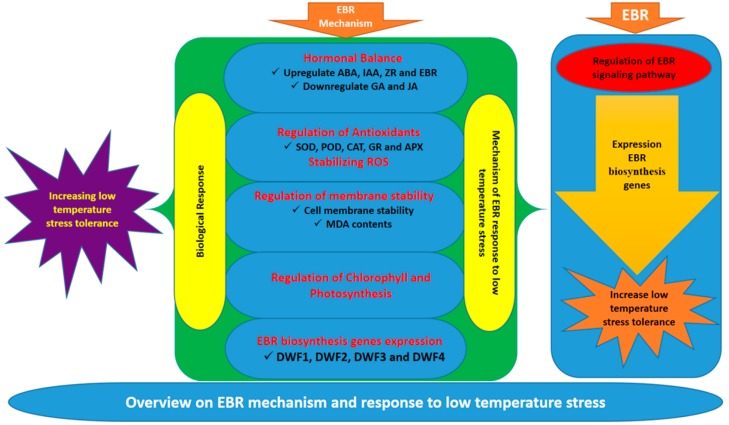
A proposed model for induction of low-temperature stress tolerance by EBR in cucumber seedlings.

The results of the present study suggest that low-temperature stress caused oxidative stress by overproduction of ROS and MDA, and decreased antioxidant enzyme activity which would otherwise damage membrane stability, reduce chlorophyll, and limit photosynthetic capacity, all of which result a significant reduction in cucumber seedling growth. Application of exogenous EBR alleviated this inhibition by improving the chlorophyll content and photosynthetic capacity levels, thus enhancing tolerance to low-temperature stress. In addition, exogenous EBR played a role in regulation of the accumulation of phytohormones, which may have a positive effect on the capability of a plant to enhance tolerance to low-temperature stress. As presented in Figure 6, endogenous hormonal balance by EBR may signal antioxidant enzymes that reduce the overproduction of ROS and lead to protection of chlorophyll and the photosynthetic machinery under low-temperature stress. Although the mechanism of EBR and phytohormones regulation has not been fully elucidated under low-temperature stress, this study has given new insights into plant resistance to low-temperature stress through EBR application and interactions with endogenous plant hormones.

## Figures and Tables

**Table 1:** Effect of EBR on chlorophyll content of cucumber seedlings under low-temperature stress.

No.	Chl a (mg/g FW)	Chl b (mg/g FW)	Carotenoid (mg/g FW)	Chl a+b (mg/g FW)	Chl a/b (mg/g FW)
NT	1.58 ± 0.02 a	0.52 ± 0.01 a	0.53 ± 0.01 a	2.12 ± 0.03 a	3.04 ± 0.10 c
CK	1.17 ± 0.01 d	0.21 ± 0.00 c	0.41 ± 0.01 b	1.38 ± 0.02 d	5.64 ± 0.17 a
EBR	1.44 ± 0.02 b	0.30 ± 0.04 b	0.49 ± 0.05 a	1.74 ± 0.05 b	4.80 ± 0.58 b
BZR	1.25 ± 0.06 c	0.22 ± 0.01 c	0.48 ± 0.02 a	1.46 ± 0.06 c	5.75 ± 0.36 a

Means in the same category followed by different letters indicate significant differences at *p* < 0.05 using the least significant difference (LSD) test. The data represent the means of four replications ± standard deviation.

**Table 2:** Effect of EBR on photosynthesis under low temperature stress in cucumber seedlings.

No.	Pn (μmol·m^−2^·s^−1^)	Gs (mmol·m^−2^·s^−1^)	Ci (μmol·mol^−1^)	Tr (mmol·m^−2^·s^−1^)
NT	9.09 ± 0.66 a	0.17 ± 0.01 a	207.25 ± 26.69 a	3.74 ± 0.16 a
CK	6.89 ± 0.25 b	0.13 ± 0.01 b	147.60 ± 11.30 b	2.10 ± 0.22 bc
EBR	8.30 ± 0.90 a	0.17 ± 0.04 a	201.64 ± 21.44 a	3.47 ± 0.39 a
BZR	5.60 ± 0.54 b	0.11 ± 0.03 b	104.26 ± 10.55 bc	2.01 ± 0.36 b

Means in the same category followed by different letters indicate significant differences at *p* < 0.05 using the LSD test. The data represent the means of four replications ± standard deviation. Pn, net photosynthetic rate; Gs, stomatal conductance; Ci, intercellular CO_2_ concentration; Tr, transpiration rate.

**Table 3:** The following primers were used in this experiment.

Primer Name	Primer Sequence (5′ to 3′)
DWF1-F	5′-CTGGGTGGACATCTTGGTTAAT-3′
DWF1-R	5′-TCGAAGGGTTTCTCTGTTTGAG-3′
DWF2-F	5′-TAACCTCACTGGCTCCATTTC-3′
DWF2-R	5′-CAATCCAGGAGCAGAGTCTTT-3′
DWF3-F	5′-TTGGTGGAGGTTCGAGATTATG-3′
DWF3-R	5′-TGTCTGGATGGCTGTCTTTAC-3′
DWF4-F	5′-GGAAAGTGCTTCCTGTGATTTC-3′
DWF4-R	5′-CTAGTTCTGAGCCAGTGCATAA-3′
Actin-F	5′-GGAAAGGACAGCTTGAATGG-3′
Actin-R	5′-GGAGAAGATCTGGCATCACAC-3′
